# Delayed extubation and hypertrophic pyloric stenosis: what are the predictive factors?

**DOI:** 10.3389/fped.2025.1540435

**Published:** 2025-06-27

**Authors:** Sabrine Ben Youssef, Syrine Laribi, Sawsen Chakroun, Maha Ben Mansour, Mariem Ben Fredj, Afef Toumi, Radhouen Ben Salah, Amine Ksia, Mongi Mekki, Mohsen Belghith, Samia Belhassen, Lassaad Sahnoun

**Affiliations:** ^1^ Department of Pediatric Surgery, Fattouma Bourguiba Hospital, Monastir, Tunisia; ^2^ Department of Anaesthesia and Intensive Care, Fattouma Bourguiba Hospital, Monastir, Tunisia

**Keywords:** hypertrophic pyloric stenosis, infant, general anesthesia, delayed extubation, metabolic alkalosis

## Abstract

**Introduction:**

Prolonged time to extubation after general anesthesia has been defined as a delay of more than 15 min from the end of surgery to tracheal extubation. This incident is frequently seen in infants operated on for hypertrophic pyloric stenosis (HPS), which can lead to inefficient use of operating rooms and delayed care for other patients.

**Aim:**

To evaluate the frequency of “delayed extubations” in infants who have received an extramucosal pyloromyotomy under general anesthesia and to identify the predictive factors of this incident.

**Methods:**

We report a retrospective and comparative study of patients operated for HPS at the pediatric surgery department of Monastir, between January 2020 and December 2023.

**Results:**

Thirty-four cases were collected. Delayed extubation occurred in 19 cases with very prolonged extubation (>60 min) in 3 cases. The mean age at diagnosis was 38.07 days for group 1 and 34.42 days for group 2. The average of operating time was 56 min for both groups. On the *χ*^2^ test, the difference between the groups was significant for metabolic alkalosis. However, the results were not significant for the other criteria (prematurity, hypotrophy, dehydration, hyponatremia, hypochloremia, hypokalemia, functional renal failure, pre-operative resuscitation time).

**Conclusions:**

These data suggest that metabolic alkalosis is predictive of delayed extubation in infants operated on for HPS under general anesthesia. The use of spinal anesthesia may be an alternative to general anesthesia but it remains a controversial issue, as there are few comparative data.

## 1 Introduction

Hypertrophic pyloric stenosis (HPS) is a frequent surgical emergency in infancy, requiring prompt diagnosis and surgical intervention. The standard treatment involves extramucosal pyloromyotomy, which is typically curative and associated with minimal long-term sequelae (1). However, perioperative management, particularly the timing of extubation, remains a challenge for anesthesiologists. This population is especially vulnerable due to the prevalence of dehydration, electrolyte disturbances (notably hypochloremia and metabolic alkalosis), and nutritional deficits.

Although most infants are full-term, prematurity can exacerbate perioperative risks, including delayed extubation, due to immature respiratory control (2, 3). Additionally, persistent metabolic imbalances despite preoperative correction may contribute to postoperative respiratory depression (4).

Therefore, it is essential to understand the factors associated with delayed extubation in this context. The objective of this study was to evaluate the incidence of delayed extubation in infants undergoing surgery for HPS and to identify the clinical and biochemical predictors associated with this incident.

## 2 Methods

### 2.1 Study design and population

We conducted a retrospective and comparative study at the Pediatric Surgery Department of Monastir between January 2020 and December 2023. All infants undergoing surgery for HPS within this timeframe were eligible for inclusion.

### 2.2 Data collection

Patient records were reviewed to extract demographic, clinical, biochemical, and perioperative data. Preoperative variables included age, gender, birth weight, gestational age, electrolyte levels, ASA score, and ultrasound findings. Perioperative data covered anesthetic technique, operative duration, and extubation time.

### 2.3 Anesthesia protocol

All patients received standardized general anesthesia with preoxygenation, rapid sequence induction, and maintenance with sevoflurane. A paraumbilical regional block with ropivacaine (2 mg/ml, 0.5 ml/kg) was administered.

### 2.4 Definition of delayed extubation

Delayed extubation was defined as the need for more than 15 min between the end of surgery and tracheal extubation. Patients were classified into two groups: Group 1 (≤15 min) and Group 2 (≥16 min).

### 2.5 Statistical analysis

Continuous variables were presented as mean ± standard deviation (SD) for normally distributed data or as median with interquartile range (IQR) for non-normally distributed data.

Comparisons of continuous variables: Performed using the Student's *t*-test (if normal distribution) or Mann–Whitney *U* test (if non-normal distribution).

Comparisons of categorical variables: Conducted using the Chi-square (Chi^2^) test or Fisher's exact test when expected counts were <5.

Multivariate analysis: Variables with *p* < 0.2 in univariate analysis were included in a binary logistic regression model to identify independent predictors of delayed extubation.

A *p*-value <0.05 was considered statistically significant.

## 3 Results

### 3.1 Study population

Thirty-four infants were included in the study. Twenty-six (76.4%) were male. Three were born prematurely, with gestational ages of thirty-four, thirty-five, and thirty-six weeks. The mean age at diagnosis was thirty-six days (range: sixteen to sixty), and the average age of vomiting onset was twenty-five days.

Sixteen infants (47.1%) were underweight. Dehydration was present in fourteen cases, with eight classified as stage 1 and six as stage 2. Initial blood tests revealed metabolic alkalosis in 24 patients, hyponatremia in 14 patients, hypochloremia in 18 patients, and renal insufficiency in 4 patients. The average thickness of the muscular layer on abdominal ultrasound was 4.76 mm, with a range of 3.9–6.5 mm.

### 3.2 Preoperative resuscitation

The duration of preoperative resuscitation varied: 12 h for 12 patients (35.3%), 24 h for 11 patients (32.4%), 48 h for 8 patients (23.5%), 72 h for 2 patients (5.9%), and 96 h for one patient (2.9%).

### 3.3 Extubation outcomes

Delayed extubation occurred in 19 cases (55.88%) with very prolonged extubation (>60 min) in 3 cases (8.82%). The mean age at diagnosis was 38.07 days for group 1 and 34.42 days for group 2. The average of operating time was 56 min for both groups.

There was no difference between the 2 groups regarding muscularis thickness. On the *χ*^2^ test, the differences between the groups were significant for metabolic alkalosis (*P* = 0.05). However, the results were not significant for the other criteria (prematurity, hypotrophy, dehydration, hyponatremia, hypochloremia, hypokalemia, functional renal failure, preoperative resuscitation time, age at the time of diagnosis, age of onset of vomiting, thickness of the muscularis, operation time) (Table 1). Metabolic alkalosis observed in our study was a pre-existing condition rather than one that developed intraoperatively or postoperatively. This condition likely contributed to respiratory depression, leading to delayed extubation. The three cases of very prolonged extubation (>60 min) were associated with significant metabolic alkalosis and electrolyte imbalances, which may have contributed to delayed respiratory recovery.

**Table 1:** Univariate comparison of demographic, clinical, and biochemical characteristics according to extubation time in infants undergoing pyloromyotomy.

Studied factors	Group 1 (15 min) *n* (%)	Group 2 (16 min) *n* (%)	*p*-value	Test used
Prematurity	2 (66.7%)	1 (33.3%)	1.00	Fisher
Hypotrophy	8 (50%)	7 (50%)	0.51	Chi^2^
Metabolic alkalosis	16 (66.7%)	8 (33.3%)	0.05	Chi^2^
Hyponatremia	7 (50%)	7 (50%)	0.56	Chi^2^
Hypochloremia	10 (55.6%)	8 (44.4%)	0.96	Chi^2^
Hypokalemia	2 (66.7%)	1 (33.3%)	1.00	Fisher
Functional renal failure	1 (25%)	3 (75%)	0.61	Fisher
Dehydration	9 (64.3%)	5 (35.7%)	0.50	Chi^2^
Preoperative resuscitation ≤24 h	13 (56.5%)	10 (43.5%)	1.00	Chi^2^
Preoperative resuscitation >24 h	6 (54.5%)	5 (45.5%)		Chi^2^
Age at diagnosis (days)	34.22 ± 6.1	38.01 ± 7.2	0.42	*t*-test
Age of vomiting onset	22.42 ± 5.3	28.00 ± 6.1	0.19	*t*-test
Muscularis thickness (mm)	4.72 ± 0.5	4.80 ± 0.6	0.75	*t*-test
Operation time (min)	56.0 ± 8.2	56.0 ± 7.9	0.99	*t*-test

In the multivariate analysis, we evaluated factors with a *p*-value threshold of <0.2, including: metabolic alkalosis, age of onset of vomiting. Our study confirms that metabolic alkalosis is an independent predictive factor for delayed extubation (*p* = 0.06). While metabolic alkalosis was corrected preoperatively, residual effects on the central respiratory drive could explain its impact on extubation time. Additionally, the binary logistic regression suggests that age of vomiting onset was not a significant predictor after adjustment (Table 2).

**Table 2:** Multivariate logistic regression identifying independent predictors of delayed extubation following surgery for hypertrophic pyloric stenosis.

Independent predictor	OR	IC 95%	*P* value
Metabolic alkalosis	4.75	0.92–24.5	0.06
Age of vomiting onset	0.69	0.90–1.02	0.19

## 4 Discussion

### 4.1 Interpretation of findings

Hypertrophic pyloric stenosis (HPS) is characterized by hypertrophy of the pyloric muscle, causing gastric outlet obstruction. Its incidence ranges from 0.9 to 5.1 per 1,000 live births (5). While delayed extubation in these patients is not widely reported in the literature, it is a phenomenon well-recognized in clinical practice. In our cohort, more than half of the infants experienced delayed extubation, and a statistically significant association was found with metabolic alkalosis.

This finding aligns with the hypothesis that metabolic disturbances particularly alkalosis affect central respiratory drive. In our study, the three patients with very prolonged extubation (>60 min) all had notable electrolyte disturbances, suggesting that residual alkalosis, even after preoperative correction, may lead to delayed recovery of spontaneous ventilation.

### 4.2 Relation to existing evidence

Several studies have reported that metabolic alkalosis impairs the ventilatory response to hypercapnia, particularly through central nervous system mechanisms (6). Cases of apnea prior to any anesthetic administration have been documented in infants with HPS (7), supporting the idea that respiratory depression can occur independently of anesthetic effects.

Polysomnographic data reinforce this interpretation. Two studies (8, 9) found significantly higher apnea–hypopnea indices preoperatively in infants with HPS, which decreased after surgery. This change is attributed to the correction of hydroelectrolytic imbalance. However, cerebrospinal fluid alkalinity may persist beyond serum correction, continuing to blunt the central response to CO_2_ and delaying extubation.

Although previous research has suggested associations between low birth weight, prematurity, and postoperative respiratory complications (10, 11), our study did not demonstrate statistically significant associations for these factors. This could be due to the small number of premature infants included in our sample (*n* = 3). Nonetheless, these variables remain clinically relevant and should be explored in larger cohorts.

The overexpression of GABAergic receptors and immature pharyngeal and brainstem pathways in preterm infants further predispose them to apnea (12). Despite these physiologic vulnerabilities, our findings indicate that metabolic alkalosis may play a more central role in delayed extubation than prematurity or low birth weight.

### 4.3 Strengths and limitations

This study presents several strengths. It is one of the few to investigate the predictors of delayed extubation in the context of pyloric stenosis using a standardized anesthetic protocol and objective time-based definitions. However, the retrospective design introduces potential selection and reporting bias.

The limited sample size (*n* = 34) reduces the power of statistical analyses, particularly for variables like prematurity. Furthermore, the variability in surgical teams and potential undocumented intraoperative factors (e.g., depth of anesthesia, opioid dosing) may confound extubation outcomes.

### 4.4 Clinical implications and future directions

Our results highlight the importance of recognizing metabolic alkalosis as a potentially modifiable risk factor for delayed extubation in infants with HPS. Thorough and early correction of electrolyte imbalances is essential, but clinicians should also be aware of residual effects on central respiratory control.

Spinal anesthesia has been proposed as an alternative approach to reduce respiratory depression in high-risk neonates. Although our study did not compare anesthesia techniques, existing literature supports its feasibility in selected cases. Future prospective, multicenter trials are needed to assess the role of spinal anesthesia and validate metabolic markers as predictors of extubation delay.

## 5 Conclusion

In conclusion, delayed extubation is common after pyloromyotomy in infants and appears to be significantly associated with preoperative metabolic alkalosis. While general anesthesia remains standard, careful metabolic optimization and exploration of alternative anesthetic techniques may improve postoperative respiratory outcomes. Further research is necessary to refine perioperative protocols in this vulnerable population.

## Data Availability

The original contributions presented in the study are included in the article/Supplementary Material, further inquiries can be directed to the corresponding author.
